# Clinical effects of interspace between the popliteal artery and capsule of the posterior knee block with multimodal analgesia for total knee arthroplasty: a systematic review and meta-analysis

**DOI:** 10.1007/s00402-023-04798-x

**Published:** 2023-02-15

**Authors:** Yongjie Qiao, Feng Li, Lvdan Zhang, Xiaoyang Song, Xinyuan Yu, Wenbin Yang, Shenghu Zhou, Haoqiang Zhang

**Affiliations:** 1 Department of Joint Surgery, The 940th Hospital of Joint Logistic Support Force of Chinese People’s Liberation Army, No. 333, Nanbinghe Road, Qilihe District, Lanzhou, 730050 Gansu China; 2 Department of Orthopedics, The 943rd Hospital of Joint Logistic Support Force of Chinese People’s Liberation Army, Wuwei, Gansu China; 3 Department of Respiratory Medicine, The 940th Hospital of Joint Logistic Support Force of Chinese People’s Liberation Army, Lanzhou, Gansu China

**Keywords:** Analgesia, Total knee arthroplasty, Pain, IPACK block, Systematic review, Meta-analysis

## Abstract

**Purpose:**

Combination of regional anaesthesia technique that is most effective in analgesia and postoperative functional outcome with the fewest complications needs investigation. Interspace between the popliteal artery and the capsule of the posterior knee block (IPACK) has been introduced clinically. We evaluated the efficacy of IPACK in combination with other nerve blocks after total knee arthroplasty.

**Methods:**

Data were obtained from PubMed, Cochrane Library, Web of Science, and Sciencedirect. Studies that compared outcomes using IPACK combined with other regional nerve blocks after total knee arthroplasty with other analgesic modalities and those which used pain scores or opioid consumption as primary or secondary outcomes were included.

**Results:**

Seventeen articles (20 trials, 1652 patients) were included. IPACK supplementation significantly reduced rest pain scores after total knee arthroplasty at postoperative hours 8–12(95%CI − 0.85 [− 1.36, − 0.34], *I*^2^ = 94%, *p = *0.001), postoperative day 1 (95% CI − 0.49 [− 0.85, − 0.14], *I*^2^ = 87%, *p = *0.006), and postoperative day 2 (95% CI − 0.28 [− 0.51, -0.05], I2 = 72%, *p = *0.02); there was no significant difference at postoperative day 3 or discharge (95% CI − 0.14 [− 0.33, 0.05], *I*^2^ = 0%, *p = *0.14). Combination treatment resulted in reduced dynamic pain scores at postoperative hours 8–12 (95%CI − 0.52 [− 0.92, − 0.12], *I*^2^ = 86%, *p = *0.01) and postoperative day 1(95% CI − 0.49 [− 0.87, − 0.11], *I*^2^ = 88%, *p = *0.01). There was no difference between postoperative day 2(95% CI − 0.29 [− 0.63, 0.05], *I*^2^ = 80%, *p = *0.09), postoperative day 3 or discharge (95% CI − 0.45 [− 0.92, 0.02], *I*^2^ = 83%, *p = *0.06). In addition, it strongly reduced postoperative opioid consumption within 24 H (95% CI − 0.76 [− 1.13, − 0.39], *I*^2^ = 85%, *p < *0.00001), 24–48 H (95% CI − 0.43 [− 0.85, − 0.01], *I*^2^ = 83%, *p = *0.04), and total opioid use (95% CI − 0.64 [− 1.07, − 0.22], *I*^2^ = 86%, *p = *0.003). Although IPACK supplementation improved timed up and go test and walking distance at postoperative day 2, there was no statistically significant difference at other time periods or obvious improvement in knee range of motion and quadriceps strength. IPACK block supplementation could shorten the length of stay (LOS) (95% CI − 0.40 [− 0.64, − 0.15], *I*^2^ = 70%, *p = *0.001) and improve patient satisfaction (95% CI 0.43 [0.01, 0.84], *I*^2^ = 87%, *p = *0.04).

**Conclusion:**

Based on these results, IPACK supplementation, in addition to standard postoperative analgesia, can be used effectively and safely to relieve early postoperative pain after total knee arthroplasty.

**Supplementary Information:**

The online version contains supplementary material available at 10.1007/s00402-023-04798-x.

## Introduction

Total knee arthroplasty (TKA) is one of the most utilized and successful procedures available to resolve end-stage knee disease and can significantly improve patients’ quality of life and knee function postoperatively. With progressive global aging, the annual worldwide rate of TKA has increased steadily over the past 2 decades [2]. However, TKA is one of the most painful procedures; therefore, adequate analgesia is a priority for orthopaedic surgeons [40]. Severe pain can prolong hospital stays, reduce patient satisfaction, and increase opioid consumption, which in turn, triggers gastrointestinal problems, cognitive dysfunction, urinary retention, pruritus, and respiratory depression. Additional complications, such as myocardial infarction and lower extremity deep vein thrombosis, may occur [10]. Adequate analgesia after TKA not only improves patient satisfaction, but also provides the foundation for postoperative functional recovery and joint mobility. Additionally, it can effectively prevent the development of chronic pain, especially for those at risk thereof. The concept of enhanced recovery after surgery (ERAS) was first introduced in 1997 by Kehlet [18], which has gradually been applied and matured in the field of orthopaedics. Regional anaesthesia (RA) could contribute further to effective analgesia for TKA and provide early functional recovery to the greatest extent possible. Therefore, RA for TKA is one of the recommended interventions in the ERAS protocol [21]. The use of multimodal analgesia should be encouraged because it not only improves joint activity, but also promotes early painless joint movement and recovery after undergoing TKA.

The complex innervation of the knee joint includes the femoral, common peroneal, anterior saphenous, tibial, and posterior obturator nerves; these serve as important targets in postoperative analgesia after TKA [30]. Subsequently, multiple regional anaesthetic modalities are often required to provide adequate postoperative analgesia. Targeting the femoral, sciatic, iliac fascia and other nerves using regional nerve blocks can achieve satisfactory early postoperative analgesia to some extent with fewer side effects than opioids; however, there is concern for the risk of potential postoperative falls due to muscular weakness, foot drop, and masking of surgically-induced peroneal nerve injury [5]. Local infiltration analgesia (LIA) does not affect the patient's movement yet does not achieve satisfactory analgesia. The adductor canal block (ACB) can provide comparable anterior knee analgesia similar to that of the femoral nerve block and preserves the main motor branch of the femoral nerve, thus maintaining balance and quadriceps strength. Complications due to poor muscle strength are avoided, however, this approach does effectively address posterior knee pain [5, 19]. Contrastingly, IPACK can provide more satisfactory posterior and lateral knee analgesia in patients after TKA, while preserving the motor function of the common peroneal nerve and tibial nerve by blocking the sensory nerve [1, 5].

Ultrasound-guided IPACK, a new regional analgesic technique, is believed to relieve posterior knee pain after TKA by targeting the articular branches which innervate the posterior aspect of the knee joint [36]. Hence, IPACK is gradually being used clinically. Whether adding IPACK to the multimodal analgesic regimen can further improve the analgesic effect after TKA needs investigation [13, 22]. There are various regional anaesthesia techniques; it is unclear which combination is the most effective in terms of analgesia and postoperative functional outcomes in nerve blocks with the fewest complications. We aimed to summarize all the RCTs and prospective studies recently published on IPACK for postoperative analgesia after TKA, and to compare the IPACK with or without other nerve blocks and other analgesic methods (FNB, ACB, LIA, etc.). Furthermore, we sought to evaluate its status and value in perioperative management after TKA, by evaluating its impact on postoperative drug use, joint mobility, patient satisfaction, joint function scores and safety profile.

## Main text

### Systematic review

This systematic review and meta-analysis adhered to the Preferred Reporting Items for Systematic Reviews and Meta-Analyses (PRISMA) guidelines. We performed a systematic search of various electronic databases (i.e.: PubMed, Cochrane Library, Web of Science, and Sciencedirect) for relevant articles from inception to July 20, 2022, without language and date restrictions. Broad MeSH terms and Boolean operators were selected for each database search; the following search terms were used: (total knee replacement OR total knee arthroplasty OR TKR OR TKA) AND (IPACK OR interspace between the popliteal artery and the capsule of the posterior knee) AND (random OR prospective OR blind).

**Fig. 1:**
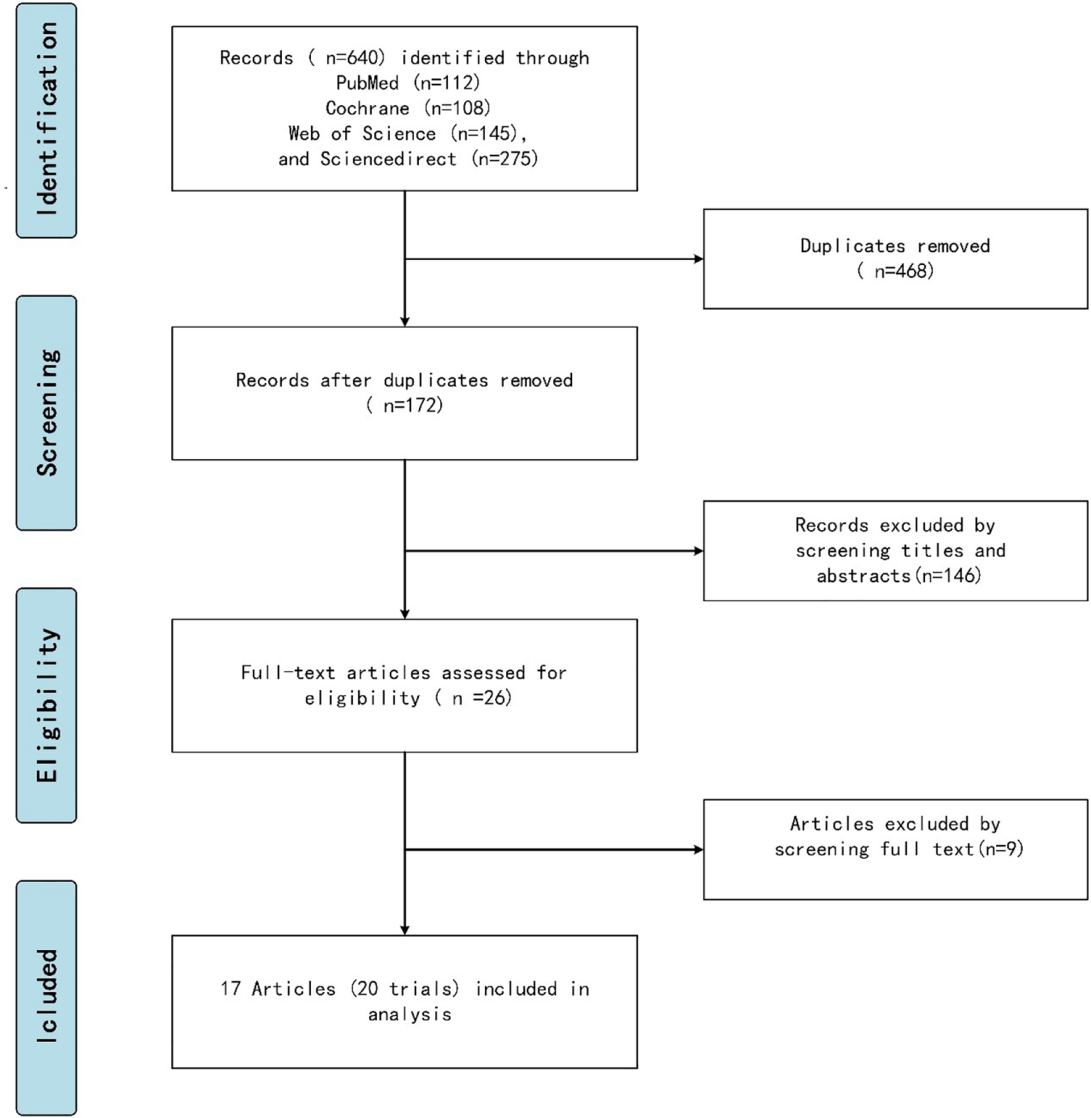
Flow diagram of the review and selection of studies

Inclusion criteria: All RCTs and prospective studies that compared outcomes using IPACK combined with other regional nerve blocks after TKA compared with other analgesic modalities were included. Studies with pain scores or opioid consumption as primary or secondary outcomes were also included. Exclusion criteria: Non-peer reviewed publications, certain study designs (observational studies, case reports, case series, review articles, letters to the editor), and non-human trials were excluded. Two authors independently selected abstracts as well as full-text articles from the above listed databases using the aforementioned search strategies, and a third author adjudicated discrepancies. The article selection process is illustrated in Fig. 1.

### Data extraction and analysis

The following data were extracted: (1) demographic and clinical information of the patients(including age, number of IPACK cases, number of control cases; (2) visual analog scale (VAS) or numerical rating scale (NRS) scores for rest and dynamic status at PO hours 8–12, POD1, 2, 3, and discharge; (3) opioid consumption at 24 h, 24–48 h, and total opioid consumption; (4) LOS and patient satisfaction; (5) TUG, walking distance, ROM, and quadriceps strength; (6) location of IPACK (proximal or distal); (7) anaesthetic doses for IPACK and other nerve blocks; (8) other nerve blocking methods; and (9) complications. Images and tables in the text were evaluated and analysed in order to extract the required data. For each included study, two reviewers extracted all relevant data independently, and any disagreement was resolved by a third reviewer.

### Quality of evidence and risk of bias assessment

**Fig. 2:**
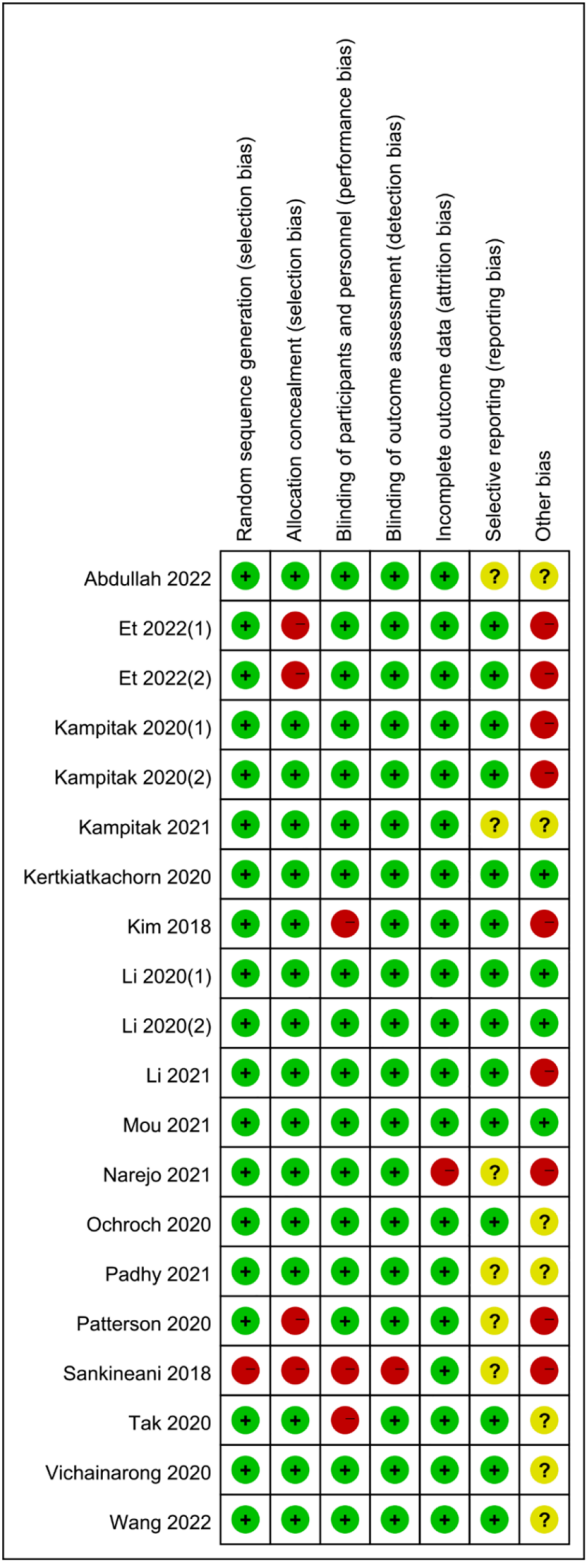
Cochrane collaboration risk of bias summary: evaluation of bias risk items for each included study. Green circle, low risk of bias; red circle, high risk of bias; yellow circle, unclear risk of bias

The quality of evidence and risk of bias for all RCT trials were assessed for methodological quality using the Cochrane Collaboration's Risk of Bias Tool by two of the researchers. Any disagreements were adjudicated by a third researcher. Risk of bias was graded as low, high, or unclear as represented in Fig. 2 as follows: green circle, low risk of bias; red circle, high risk of bias; yellow circle, unclear risk of bias. RCTs are considered high-quality literature; however, the level of evidence may be downgraded due to risk of bias, variability, imprecision, and publication bias. The risks of bias are presented in the Fig. 2.

### Statistical analysis

All analyses were conducted using the Stata software (RevMan version 5.3.5, The Nordic Cochrane Centre, The Cochrane Collaboration 2014, Copenhagen, Denmark). The continuous variable median (and interquartile range (IQR)) was estimated by the method described by McGrath et al. [26], and converted into mean ± standard deviation (SD) for inclusion in the statistical analysis. The data was expressed as weighted or standardized mean difference (WMD or SMD). We calculated the I^2^ coefficient to assess heterogeneity with the following predetermined limits: low < 50%, moderate 50–74%, and high > 75%; and *P* ≥ 0.05 and I^2^ < 50% indicating no statistical heterogeneity between studies. A random-effects model was applied in circumstances of moderate or high heterogeneity; otherwise, a fixed-effects model was employed. If there was significant heterogeneity in the included RCTs, such data were considered unsuitable.

### Results

A total of 17 publications with 1652 patients were included [1, 9, 15–17, 19, 20, 22, 23, 27, 28, 31–33, 35, 37, 41, 43], consisting of 16 RCTs and one prospective control trial. The trial characteristics are represented in Table 1. All studies investigated the analgesic efficacy of IPACK in patients undergoing TKA only.

**Table 1:** Trial characteristics

First author et al., year, study type	Group (N)	Gender (M/F)	Age (mean)	Local anesthetic used	IPACK location	Intraop anesthesia	Preop analgesia	Postop MMA	Outcome measures
Sankineani et al. [35], 2018, prospective control trial	IPACK + ACB (60)	38/22	66.6	15 ml 0.2% ropiv (IPACK) 20 ml 0.2% ropiv (ACB)	Proximal	Spinal	Celecoxib 200 mg Gabapentin 300 mg	Yes	VAS pain scores ROM Walking distance
	ACB (60)	42/18	63.3						
Kim et al. [20], 2018, RCT	IPACK + ACB + PAI (43)	20/23	63.8	25 ml 0.25% bupiv (IPACK) 15 ml 0.25% bupiv (ACB) 30 ml 0.25% bupiv w/epi (deep PAI) 20 ml 0.25% bupiv (superficial PAI) 30 ml 0.5% bupiv w/epi (deep PAI) 20 ml 0.25% bupiv (superficial PAI)	Proximal	Spinal	Meloxicam 15 mg (7.5 mg, age > 75) Oxycodone 10 mg	Yes	NRS pain scores Opioid use Walking distance Satisfaction
	PAI (43)	13/30	67.1						
Patterson et al. [33], 2020, RCT	IPAC + CACB (35)	14/21	67 (median)	20 ml 0.25% ropiv w/epi (IPACK) 20 ml 0.25% ropiv w/epi + 8 ml/h 0.2% ropiv for 48 h (CACB) 2 ml 0.9% saline (sham + IPACK)	Proximal	General and spinal	Pregabalin 150 mg (75 mg, age > 70)	Yes	VAS pain score Opioid use Walking distance LOS
	CAC + sham IPACK (34)	13/21	68 (median)						
Kampitak et al. [15], 2020 (1), RCT	IPACK + CACB (33)	7/26	68.6	20 ml 0.25% levobupiv w/epi (IPACK) 15 ml 0.25% levobupiv + 5 ml/h 0.15% levobupiv (CACB) 15 ml 0.25% levobupiv (TNB)	Proximal	Spinal	Paracetamol 650 mg	Yes	NRS pain scores Opioid use TUG ROM LOS Quadriceps strength Satisfaction
	CACB + TNB (32)	4/28	68.8						
Kampitak et al. [15], 2020 (2), RCT	IPACK + CACB (33)	6/27	69.9	20 ml 0.25% levobupiv w/epi (IPACK) 15 ml 0.25% levobupiv + 5 ml/h 0.15% levobupiv (cACB) 15 ml 0.25% levobupiv (TNB)	Distal	Spinal	Paracetamol 650 mg	Yes	NRS pain scores Opioid use TUG ROM LOS Quadriceps strength Satisfaction
	CACB + TNB (32)	4/28	68.6						
Li et al. [22], 2020 (1), RCT	IPACK + ACB + LFCNB (50)	17/33	66.3	20 ml 0.2% ropiv w/epi (IPACK) 20 ml 0.2% ropiv w/epi (ACB) 10 ml 0.2% ropiv w/epi (LFCNB)	Distal	General	Loxoprofen 60 mg	Yes	VAS pain scores Opioid use ROM TUG LOS Walking distance Quadriceps strength
	ACB + LFCNB (50)	18/32	66.4						
Li et al. [22], 2020 (2), RCT	IPACK + ACB (50)	10/40	66.8	20 ml 0.2% ropiv w/epi (IPACK) 20 ml 0.2% ropiv w/epi (ACB)	Distal	General	Loxoprofen 60 mg	Yes	VAS pain scores Opioid use ROM TUG LOS Walking distance Quadriceps strength
	ACB (50)	19/31	65.6						
Kertkiatkachorn et al. [19], 2020, RCT	IPACK + ACB + postoperative CACB + sham PAI (34)	29/4	70.6	20 ml 0.25% levobupiv w/epi (IPACK) 20 ml 0.25% levobupiv w/epi (ACB) 5 ml/h 0.15% levobupiv for 60 h (postoperative CACB) 80 ml 0.125% levobupiv w/epi (PAI) 20 ml 0.9% saline (sham IPACK) 80 ml 0.9% saline (sham PAI) 20 ml 0.9% saline (sham ACB)	Distal	Spinal	Acetaminophen 750 mg celecoxib 400 mg	Yes	VAS pain scores Opioid use TUG ROM LOS Satisfaction Quadriceps strength
	PAI + postoperative CACB + sham IPACK + sham ACB (35)	29/5	68.7						
Vichainarong et al. [41], 2020, RCT	IPACK + CACB + PAI (33)	4/29	70.7	20 ml 0.25% levobupiv w/epi (IPACK) 20 ml 0.25% levobupiv + 5 ml/h 0.15% levobupiv for 60 h (CACB) 80 ml 0.125% levobupiv w/epi (PAI) 5 ml 0.9% saline (sham IPACK)	Distal	Spinal	Acetaminophen 650 mg celecoxib 400 mg	Yes	NRS pain scores Opioid use TUG ROM LOS Satisfactio Quadriceps strength
	CACB + PAI + sham IPACK (32)	5/27	68.7						
Ochroch et al. [31], 2020, RCT	IPACK + CACB (60)	26/34	67.7	20 ml 0.5% ropiv (IPACK) 18 ml 0.5% ropiv + 8 ml/h 0.2% ropiv for 48 h (CACB)	Distal	General and spinal	Acetaminophen 1000 mg gabapentin 300 mg celecoxib 200 mg	Yes	APS-POQ-R pain scores Opioid use TUG Walking distance Satisfaction
	CACB + sham IPACK (59)	24/35	65.6						
Tak et al. [37], 2020, RCT	IPACK + ACB (56)	27/29	65.5	20 ml 0.2% ropiv (IPACK) 20 ml 0.2% ropiv (ACB)	Proximal	Spinal	Celecoxib 200 mg gabapentin 300 mg	Yes	VAS pain scores Opioid use TUG ROM Walking distance
	ACB (58)	21/37	64.1						
Kampitak et al. [17], 2021, RCT	IPACK + ACB + AFCNB + sham PAI (34)	2/32	70.0	20 ml 0.25% levobupiv w/epi (IPACK) 20 ml 0.25% levobupiv w/epi (ACB) 20 ml 0.25% levobupiv w/epi (AFCNB) 80 ml 0.1875% levobupiv w/epi (PAI) 80 ml 0.9% saline (sham PAI) 20 ml 0.9% saline (sham IPACK) 20 ml 0.9% saline (sham ACB) 20 ml 0.9% saline (sham AFCNB)	Distal	Spinal	Acetaminophen 650 mg	Yes	NRS pain scores Opioid use ROM LOS Satisfaction
	PAI + sham IPACK + sham ACB + sham AFCNB (34)	1/33	73.2						
Narejo et al. [28], 2021, RCT	IPACK + ACB (40)	10/30	64.3	20 ml 0.25% bupiv (IPACK) 20 ml 0.25% bupiv (ACB) 60 ml 0.167% bupiv w/epi (PAI)	Distal	Spinal	NA	Yes	NRS pain scores TUG ROM LOS
	PAI + ACB (40)	7/33	64.2						
Mou et al. [27], 2021, RCT	IPACK + ACB (40)	8/32	64.4	20 ml 0.25% ropiv w/epi (IPACK) 20 ml 0.25% ropiv w/epi (ACB) 20 ml 0.9% saline (sham IPACK)	Distal	General	Celecoxib 200 mg	Yes	VAS pain scores Opioid use TUG ROM Quadriceps strength
	ACB + sham IPACK (40)	6/34	66.4						
Padhy et al. [32], 2021, RCT	IPACK + ACB (41)	23/18	62.6	15 ml 0.25% ropiv (IPACK) 15 ml 0.25% ropiv (ACB) 15 ml 0.25% ropiv (SPANK)	Proximal	Spinal	NA	Yes	NRS pain scores Opioid use Satisfaction Quadriceps strength
	ACB + SPANK (41)	22/19	64.5						
Li et al. [23], 2021, RCT	IPACK + FTB (40)	15/25	67.8	20 ml 0.3% ropiv w/morphine (IPACK) 10 ml 0.3% ropiv w/morphine (FTB) 100 ml. 0.15% ropiv w/morphine (PAI)	Distal	General	Celecoxib 200 mg	Yes	VAS pain scores Opioid use ROM LOS Quadriceps strength
	PAI (40)	18/22	70.8						
Abdullah et al. [1], 2022, RCT	IPACK + ACB (40)	19/21	60.1	30 mL 0.25% bupiv (IPACK) 20 mL 0.25% bupiv (ACB)	Proximal	Spinal	NA	Yes	VAS pain scores Opioid use TUG Quadriceps strength Satisfaction
	ACB (40)	16/24	60.8						
Et et al. [9], 2022 (1), RCT	IPACK + ACB (35)	12/23	68.5	20 ml 0.25% bupiv (IPACK) 20 ml 0.5% bupiv (ACB) 40 ml 0.25% bupiv w/morphine (PAI)	Distal	Spinal	acetaminophen 1000 mg Diclofenac sodium 75 mg	Yes	NRS pain score Opioid use TUG ROM LOS Satisfaction
	ACB + PAI (35)	14/21	71.1						
Et et al. [9], 2022 (2), RCT	IPACK + ACB (35)	12/23	68.5	20 ml 0.25% bupiv (IPACK) 20 ml 0.5% bupiv (ACB)	Distal	Spinal	acetaminophen 1000 mg Diclofenac sodium 75 mg	Yes	NRS pain scores Opioid use TUG ROM LOS Satisfaction
	ACB (35)	16/19	67.1						
Wang et al. [43], 2022, RCT	IPACK + CACB (35)	7/28	66.5	20 ml 0.25% ropiv w/epi (IPACK) 15 ml 0.5% ropiv + 5 ml/h 0.2% ropiv for 72 h (CACB) 0.9% saline subcutaneously (sham IPACK)	Distal	General	NA	Yes	VAS pain scores Opioid use ROM Walking distance Satisfaction
	CACB + sham IPACK (35)	6/29	64.2						

*N* number; *M* male; *F* female; *MMA* multimodal analgesia; *IPACK* interspace between the popliteal artery and the capsule of the posterior knee block; *ACB* adductor canal block; *PAI* periarticular injections; *CACB* continuous adductor canal block; *FTB* femoral triangle block; *LFCNB* lateral femoral cutaneous nerve block; *SPANK* sensory posterior articular nerves of the knee; *AFCNB* anterior femoral cutaneous nerve block; *RCT* randomized control trial; *VAS* visual analog scale; *NRS* numerical rating scale; *TUG* timed up and go test; *ROM* range of movement; *LOS* length of stay; *ropiv* ropivacaine; *bupiv* bupivacaine; *levobupiv* levobupivacaine; *w/epi* with epinephrine

#### Rest pain scores

**Fig. 3:**
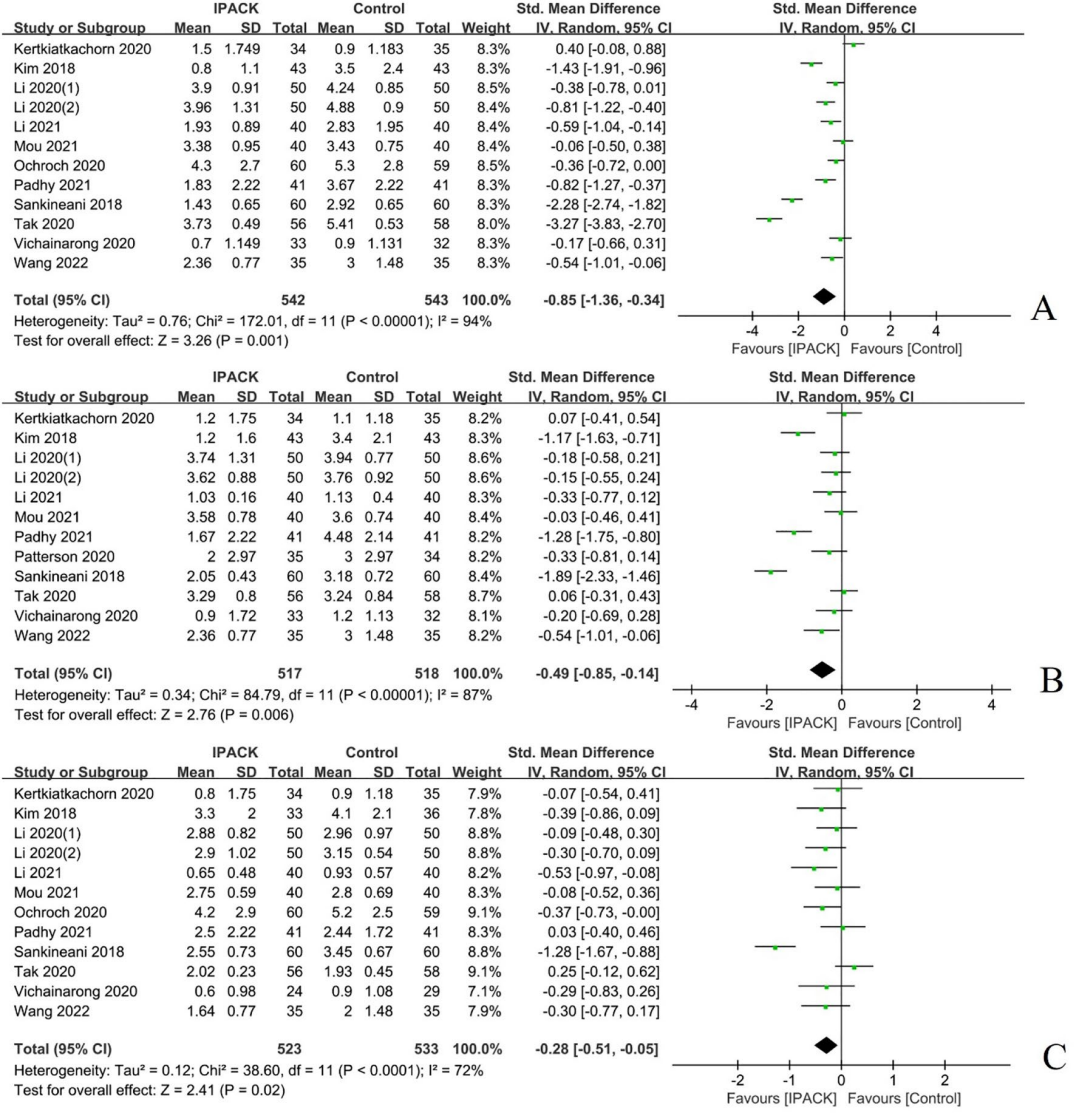
Rest pain scores after TKA with IPACK block supplementation. **A** at 8–12 postoperative hours; **B** at POD1; **C** at POD2. *TKA* total knee arthroplasty; *IPACK* interspace between the popliteal artery and the capsule of the posterior knee; *POD* postoperative day

Twelve studies (*n = *1154) reported on the total rest pain scores after TKA with IPACK supplementation [19, 20, 22, 23, 27, 31–33, 35, 37, 41, 43]. When compared with a control group, IPACK supplementation was found to reduce rest pain scores at 8–12 h postoperatively, with a mean difference (95% CI − 0.85 [− 1.36, − 0.34], *I*^2^ = 94%, *p = *0.001) as well as POD1 (95% CI − 0.49 [− 0.85, − 0.14], *I*^2^ = 87%, *p = *0.006), and POD2 (95% CI − 0.28 [− 0.51, − 0.05], *I*^2^ = 72%, *p = *0.02);. However, rest pain scores on POD3 or discharge (95% CI − 0.14 [− 0.33, 0.05], *I*^2^ = 0%, *p = *0.14) did not achieve statistical significance (Fig. 3, Table 2).

**Table 2:** Primary outcomes of TKA with IPACK block supplementation

Outcomes	Number of trials	Total number of participants		WMD or SMD, [95% CI]	*p* value for overall effect	Heterogeneity			Model
						Tau^2^	*χ*^2^	*I*^2^ (%)	
		IPACK	Control						
Rest pain scores at specific time points									
POH 8–12	12	542	543	− 0.85 [− 1.36, − 0.34]	0.001	0.76	172.01	94	Random
POD1	12	517	518	− 0.49 [− 0.85, − 0.14]	0.006	0.34	84.79	87	Random
POD2	12	523	533	− 0.28 [− 0.51, − 0.05]	0.02	0.12	38.60	72	Random
POD3 or discharge	5	215	215	− 0.14 [− 0.33, 0.05]	0.14	NA	3.98	0	Fixed
Dynamic pain scores at specific time points									
POH 8–12	9	366	366	− 0.52 [− 0.92, − 0.12]	0.01	0.32	56.20	86	Random
POD1	12	495	493	− 0.49 [− 0.87, − 0.11]	0.01	0.40	94.41	88	Random
POD2	9	347	356	− 0.29 [− 0.63, 0.05]	0.09	0.21	40.00	80	Random
POD3 or discharge	5	215	215	− 0.45 [− 0.92, 0.02]	0.06	0.24	23.30	83	Random
Opioid consumption									
Within 24 h	10	402	400	− 0.76 [− 1.13, − 0.39]	< 0.00001	0.30	58.13	85	Random
24–48 h	7	274	273	− 0.43 [− 0.85, − 0.01]	0.04	0.26	34.89	83	Random
Total opioid consumption	8	341	343	− 0.64 [− 1.07, − 0.22]	0.003	0.32	51.16	86	Random

*POH* postoperative hour(s); *POD* postoperative day; *IPACK* interspace between the popliteal artery and capsule of the posterior knee block; *TKA* total knee arthroplasty; *WMD* weighted mean difference; *SMD* standardized mean difference; *CI* confidence interval

#### Dynamic pain scores

**Fig. 4:**
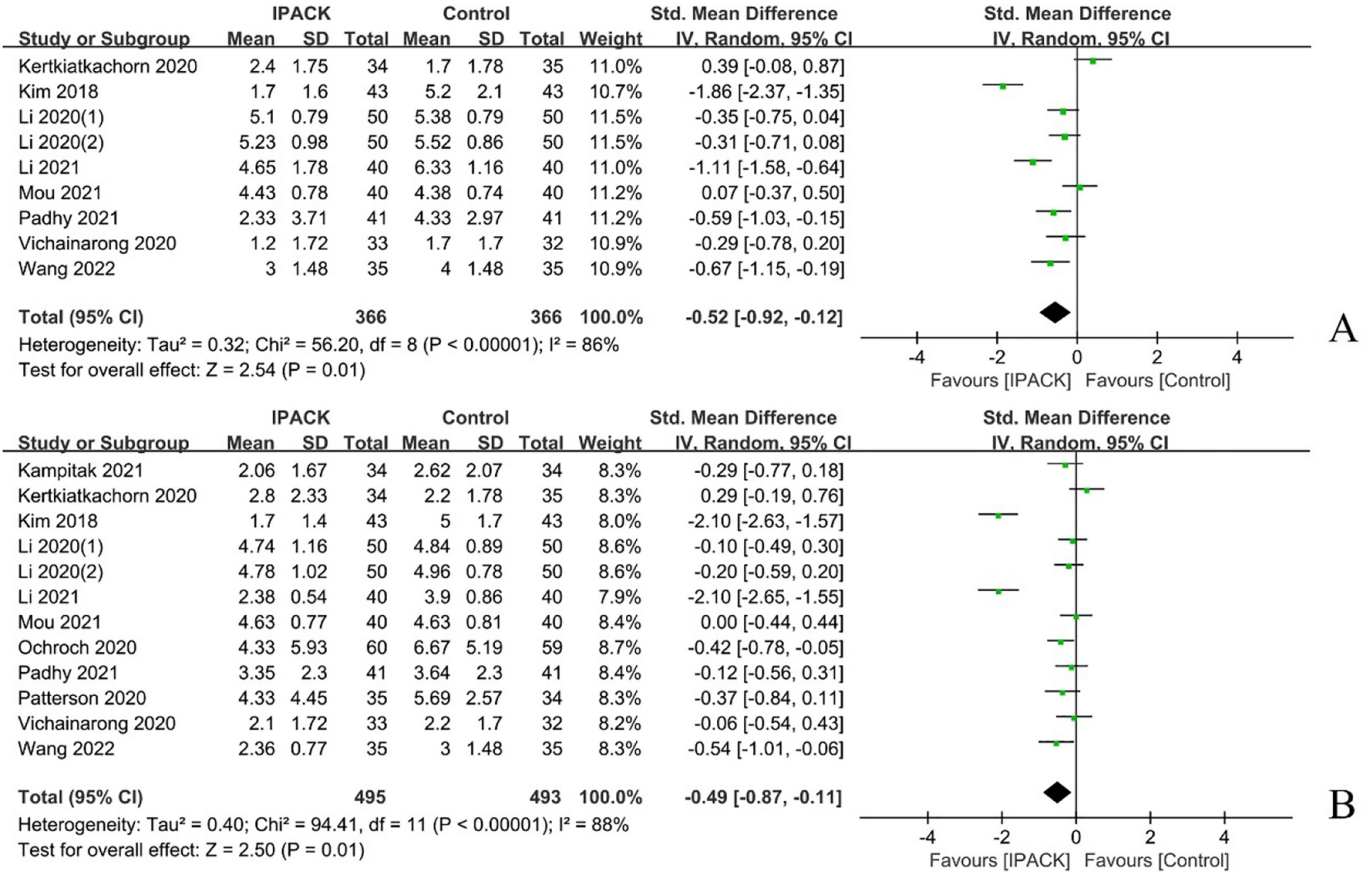
Dynamic pain scores after TKA with IPACK block supplementation. **A** at 8–12 postoperative hours; **B** POD1. *TKA* total knee arthroplasty; *IPACK* interspace between the popliteal artery and the capsule of the posterior knee; *POD* postoperative day

Eleven studies (*n = *988) were summarized regarding dynamic pain scores after TKA with IPACK supplementation [17, 19, 20, 22, 23, 27, 31–33, 41, 43]. The meta-analysis firmly indicated the analgesic benefit of IPACK at 8–12 h postoperatively (95% CI − 0.52 [− 0.92, − 0.12], *I*^2^ = 86%, *p = *0.01) and POD1 (95% CI − 0.49 [− 0.87, − 0.11], I^2^ = 88%*, p = *0.01) compared with a control group; however, the results in favour thereof on POD2 (95% CI − 0.29 [− 0.63, 0.05], *I*^2^ = 80%, *p = *0.09) and POD3 or discharge (95% CI − 0.45 [− 0.92, 0.02], *I*^2^ = 83%, *p = *0.06) with IPACK supplementation, did not demonstrate a statistically significant difference between studies (Fig. 4, Table 2).

#### Opioid consumption

**Fig. 5:**
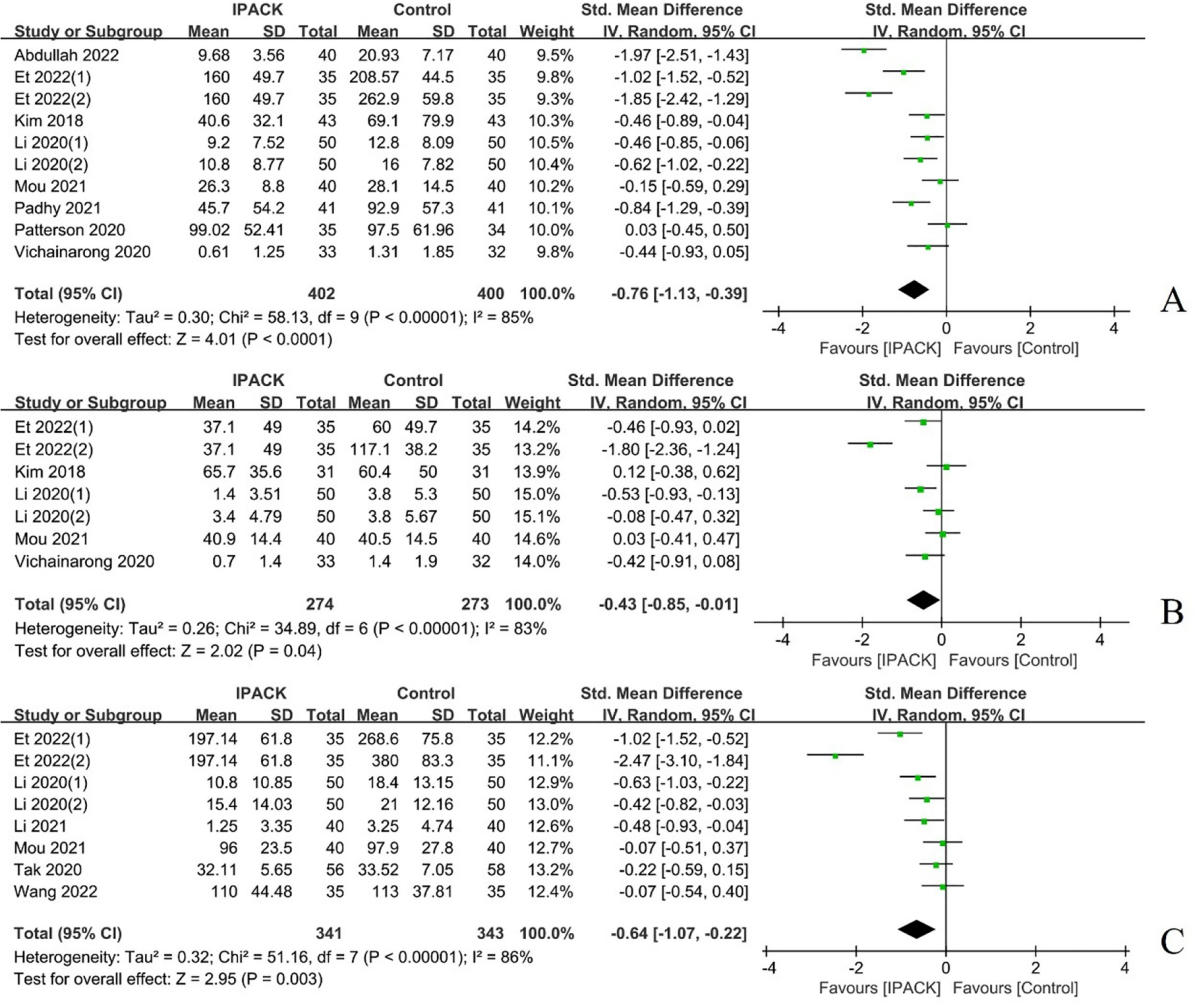
The postoperative opioid consumption after TKA with IPACK block supplementation. **A** postoperatively at 24 h; **B** at 24–48 h postoperatively; **C** total opioid consumption. *TKA* total knee arthroplasty; *IPACK* interspace between the popliteal artery and the capsule of the posterior knee block

A total of ten studies (*n = *1066) characterized postoperative opioid consumption after TKA with IPACK supplementation [1, 9, 20, 22, 27, 32, 33, 37, 41, 43], which demonstrated a significant reduction in postoperative opioid consumption at 24 h (95% CI − 0.76 [− 1.13, − 0.39], *I*^2^ = 85%, *p < *0.00001) and 24–48 h (95% CI − 0.43 [− 0.85, − 0.01], *I*^2^ = 83%, *p = *0.04); and total opioid use (95% CI − 0.64 [− 1.07, − 0.22], *I*^2^ = 86%, *p = *0.003) (Fig. 5, Table 2).

#### Length of stay and satisfaction

**Fig. 6:**
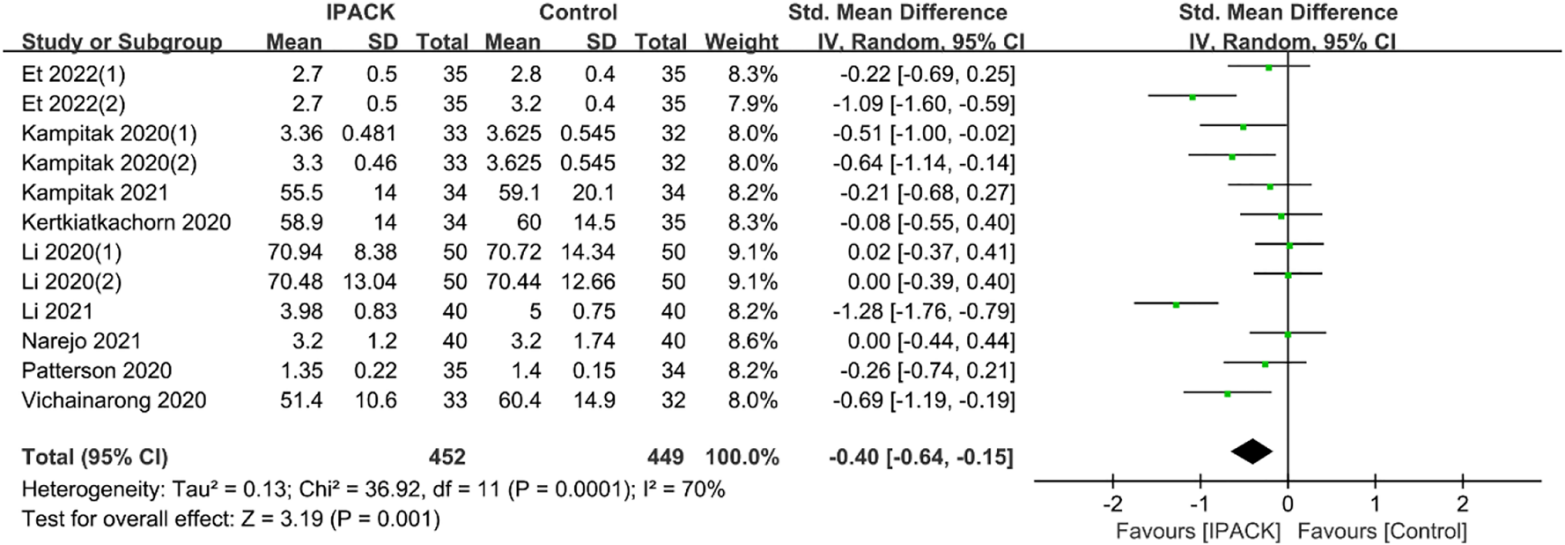
LOS after TKA with IPACK block supplementation. *LOS* length of stay; *TKA* total knee arthroplasty; *IPACK* interspace between the popliteal artery and the capsule of the posterior knee

**Fig. 7:**
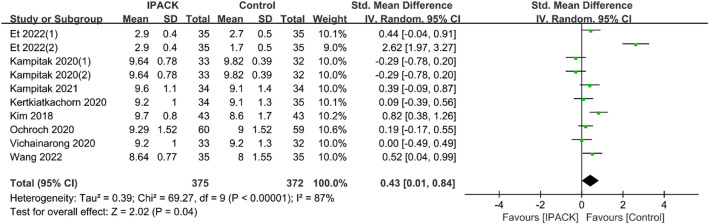
Satisfaction after TKA with IPACK block supplementation. *TKA* total knee arthroplasty; *IPACK* interspace between the popliteal artery and the capsule of the posterior knee block

Data from 11 studies (*n = *901) described the LOS, and 10 studies (*n = *747) described patient satisfaction after TKA with IPACK supplementation, respectively [9, 16, 17, 19, 20, 22, 23, 28, 31, 33, 41, 43]. The research strongly confirmed that it could shorten LOS (95% CI − 0.40 [− 0.64, − 0.15], *I*^2^ = 70%, *p = *0.001) and improve patient satisfaction (95% CI 0.43 [0.01, 0.84], *I*^2^ = 87%, *p = *0.04) (Figs. 6 and 7).

#### Physical examinations findings and complications

Although IPACK supplementation improved TUG and walking distance at POD2, there was no statistically significant difference at other time points (Table 3), nor an obvious advantage in ROM of the knee and quadriceps strength at any time points (Table 2). Among the complications assessed by the included trials, no statistically significant differences were found (Table 3).

**Table 3:** Secondary outcomes of TKA with IPACK block supplementation

Outcome	Number of trials	Total number of participants		WMD or SMD [95% CI]	*p* value for overall effect	Heterogeneity			Model
						Tau^2^	*χ*^2^	*I*^2^ (%)	
		IPACK	Control						
TUG test at specific time points									
POD1	10	380	376	− 0.08 [− 0.22, 0.07]	0.30	NA	16.21	44	Fixed
POD2	9	345	343	− 0.24 [− 0.39, − 0.09]	0.002	NA	12.28	35	Fixed
POD3 or discharge	6	262	260	− 0.06 [− 0.24, 0.11]	0.48	NA	7.8	36	Fixed
ROM at specific time points									
POD1	11	419	418	2.28 [− 0.06, 4.62]	0.06	8.99	27.57	64	Random
POD2	12	479	478	2.80 [− 0.54, 6.14]	0.10	29.07	82.89	87	Random
POD3 or discharge	8	337	337	1.63 [− 1.79, 5.06]	0.35	20.68	55.76	87	Random
Walking distance at specific time points									
POD1	6	272	270	0.08 [− 0.09, 0.25]	0.34	NA	7.63	34	Fixed
POD2	6	280	283	0.24 [0.07, 0.41]	0.005	NA	8.86	42	Fixed
POD3 or discharge	4	195	195	0.29 [− 0.10, 0.67]	0.15	0.11	11.09	73	Random
Quadriceps strength at specific time points									
POD1	8	310	305	0.05 [− 0.13, 0.23]	0.60	0.03	17.00	59	Random
POD2	7	269	265	0.08 [− 0.12, 0.28]	0.43	0.04	17.06	65	Random
POD3 or discharge	5	195	190	0.06 [− 0.25, 0.37]	0.69	0.08	36.63	89	Random
LOS	12	452	449	− 0.40 [− 0.64, − 0.15]	0.001	0.13	36.92	70	Random
Satisfaction	10	375	372	0.43 [0.01, 0.84]	0.04	0.39	69.27	87	Random

*POH* postoperative hour(s); *POD* postoperative day; *IPACK* interspace between the popliteal artery and capsule of the posterior knee block; *TKA* total knee arthroplasty; *WMD* weighted mean difference; *SMD* standardized mean difference; *CI* confidence interval

## Discussion

Considering the changing healthcare environment that emphasizes early activity and discharge after TKA, we aimed to determine whether using IPACK combined with other nerve blocks for patients after TKA allows early activity and reduces LOS while providing adequate pain relief. We found that IPACK combined with other nerve blocks could effectively relieve early pain (within 2 days after surgery) in patients after TKA, particularly rest pain at 8–12 h postoperatively, yet there was no statistically significant difference at POD3 or discharge. Dynamic pain scores at 8–12 h PO and POD1 demonstrated statistically significant differences, but none at POD2, POD3 or discharge. There were differences in opioid consumption with IPACK combined with other nerve blocks at 24–48 h PO, especially within 24 h PO. Moreover, total opioid consumption was shown to differ significantly between groups. Additionally, IPACK combined with other nerve blocks could shorten LOS and improve patient satisfaction. Interestingly, the TUG and walking distance were statistically different at POD2 but not at other time points. In our study, the ROM, quadriceps strength and complications did not correlate significantly with the addition of IPACK.

Postoperative TKA pain affects patients' functional recovery and can cause patients to refuse surgery due to fear. Therefore, better pain control after TKA is a priority for orthopaedists. Recently, ERAS and multimodal analgesia have contributed greatly to postoperative analgesia after TKA. There is an urgent need for new analgesic models which reduce complications and postoperative analgesia usage without affecting postoperative muscle strength. Compared to epidural analgesia, periarticular injections, and peripheral nerve blocks (PNB), such as the adductor canal (ACB) and saphenous nerve blocks (SNB), are widely accepted and used in multimodal analgesic protocols due to their ability to reduce opioid consumption and motor protection in patients undergoing TKA. These nerve blocks provide similar analgesia to epidural analgesia, with improved mobility and fewer complications, such as lower blood pressure and urinary retention [11, 34].

Femoral and sciatic nerve blocks are considered effective in relieving postoperative pain immediately after primary TKA and have been shown to reduce opioid use [5, 38]. However, mobility remains affected until the block wears off, which is detrimental to the patient's early functional rehabilitation of the knee. Moreover, periarticular injection and peripheral nerve block cannot achieve effective analgesia due to incomplete blockade, especially of the posterior aspect of the knee joint [19, 35]. There was no clear difference in the analgesic effect of ACB compared with FNB; likely, as most nerves in the adductor canal are sensory nerves that innervate the knee joint. FNB decreases quadriceps strength and causes corresponding complications [5, 42]. It is encouraging that IPACK has been gradually applied to analgesia after TKA in recent years, with good results [8, 20].

Ultrasound-guided IPACK targets the branching nerves which innervate the posterior aspect of the knee joint. However, the extent of injection spread and the number of affected branches remains unknown. A cadaveric study showed that latex injections spread to the middle knee artery, which usually accompanies the articular branch of the tibial nerve [29]. Another study compared the distribution of methylene blue in the proximal versus distal approach by performing an ultrasound-guided IPACK, which resulted in the nerves of the anterior external capsule of the knee joint, the anterior branch of the common peroneal nerve, the superior lateral knee nerve and the posterior capsule of the joint becoming more easily stained [39]. These studies showed that after IPACK blockade, the drug spreads to the articular branches of the common peroneal and tibial nerves, and obturator nerve, which are responsible for innervating the posterior knee capsule.

When performing posterior capsule release, TKA can cause posterior knee pain due to popliteal soft tissue debridement and cyst excision. As a result, substantial pain occurs, which is usually most intense within 24 h of a TKA and can lead to a variety of complications [12]. This study shows that IPACK + ACB greatly improved pain within 48 h after TKA compared to ACB alone; ROM on postoperative POD 2 and walking distance on POD 3 also increased, but not all functional outcomes improved [35, 37]. A RCT found that adding IPACK and ACB to periarticular injection (PAI) improved analgesia after TKA compared to PAI alone and can effectively reduce dynamic and rest NRS pain scores at POD 1 [20]. Another study showed that ACB + IPACK reduced pain within 8 h postoperatively compared with ACB alone; however, the small statistical benefit of adding IPACK to ACB is unlikely to be clinically significant [27]. Similarly, a study concluded that the IPACK + ACB group had significantly lower dynamic NRS scores at 48 h postoperatively than the PAI + ACB and ACB groups and can effectively reduce opioid consumption [9]. Another study also showed ACB + IPACK offers better analgesia, less opioid consumption and better patient satisfaction with comparable rehabilitation parameters in the immediate postoperative period after TKA, compared to ACB with sensory posterior articular nerves of the knee (SPANK) block [32]. Furthermore, receiving ACB + IPACK and lateral femoral cutaneous nerve block (LFCNB) had a longer analgesic effect than ACB combined with IPACK, ACB combined with LFNCB and ACB only [22]. It has also been shown that the IPACK alone only reduces the incidence of posterior knee pain at 6 h postoperatively but is less effective for anterolateral pain [31]. In contrast to these results, IPACK + LIA + continuous adductor canal block (CACB) did not result in better analgesia than LIA + CACB [41]. The results of this study showed that IPACK combined with other nerve blocks was effective in relieving early pain in patients after TKA, which supports the use of IPACK blockade supplementation with ACB as a motion-preserving RA technique in knee surgery, resulting in better levels of analgesia.

The goal of postoperative rehabilitation after TKA is optimal pain control with minimal opioid requirements, and better motor function preservation. Although opioids can be used for severe pain and are commonly used for analgesia after TKA, long-term opioid use may increase the risk of TKA revision in the first year and predisposes to more complications, such as nausea and vomiting [3]. Additional concerns about substance abuse and addiction, with an emphasis on reducing postoperative opioid consumption, are particularly important for patients undergoing TKA. Femoral Triangle Block (FTB) + IPACK provided excellent early postoperative analgesia and significantly reduced morphine and narcotic dosage compared to FTB block alone [23]. A study found that IPACK + ACB largely reduced postoperative opioid consumption compared to ACB alone [1]. Similarly, Kim et al. also found that IPACK + ACB + PAI resulted in less opioid use after surgery (*p = *0.005, POD 0), less intravenous opioid use (*p < *0.001), and less need for intravenous self-administered analgesia (*p = *0.037) compared with PAI alone [20]. Furthermore, a RCT showed that IPACK alone, compared with ACB, and ACB + IPACK, was associated with the highest opioid consumption within 24 h postoperatively and during hospitalization [27]. Interestingly, a prospective study found that patients given IPACK + ACBs may require more opioids and have poorer immediate functional performance compared to PAI alone [19]; this may be related to the small sample size of this particular study and the operating technique of the anaesthesiologist. The abovementioned nerve block methods (ACB, FNB, and PAI) only blocked the sensory nerves, while the motor nerves may overlap with some of the sensory nerves and still play a role in pain. Using a large sample, the results of this meta-analysis showed that IPACK combined with other regional nerve blocks greatly reduced opioid use in the early and total postoperative period after TKA; therefore, IPACK is a very promising nerve block for postoperative analgesia after TKA.

Several studies have also shown that IPACK combined with other multimodal analgesic methods can shorten LOS in TKA patients [8, 9, 41]. Similarly, studies have shown that IPACK increases the proportion of patients discharged at POD 3, possibly due to the absence of plantar numbness and the preservation of plantar flexor muscle strength [15]. However, there is no conclusive evidence that PNB can clearly shorten LOS compared to other analgesic modalities. The LOS after TKA depends on a variety of factors, including the patient's health status, ability to walk safely, overall pain control, and the patient's psychosocial circumstances. The combination of IPACK with other nerve blocks in this study remarkably reduced the LOS, which to some extent, supports the use of IPACK for analgesia after TKA. Furthermore, better postoperative analgesia, ambulation, ROM, and minimal opioid consumption and complications all improve patient satisfaction. Therefore, if the above problems are adequately addressed, patients will have greater levels of satisfaction. Previous studies have found that patients who received IPACK-inclusive analgesia demonstrated good postoperative analgesia, less opioid consumption, better ambulation, and ROM, resulting in higher patient satisfaction [20, 32]. Patient satisfaction was higher in the majority of studies with the addition of IPACK, and the results of this meta-analysis showed that the addition of IPACK improved patient satisfaction remarkably well.

Our study compared the analgesic effects of proximal and distal IPACK techniques after TKA and showed no visible difference between them. However, it is possible that the effects obtained with proximal and distal IPACKs are different, and there is uncertainty about the clinical outcome due to improper selection of the optimal block site. A cadaver study showed that the dispersion of injectables in cadavers may differ from clinical practice due to differences in tissue structure and the nature of the injectables [6]. Other factors, such as the patient's muscle contraction, patient's position, anatomical changes, needle direction and injection pressure may also affect the drug diffusion, which may also affect the analgesic effect [4]. Surprisingly, a study found that injections at or just above the level of the femoral condyle (distal technique) were more advantageous in relieving posterior knee pain [17]. Moreover, the effects may vary depending on the concentration and dose of the injected drug. However, a study showed that even if the concentration and dose of local anaesthetic drugs are lowered in peripheral nerve blocks, they remain effective, while increasing the concentration and dose increases the risk of systemic drug toxicity [24]. Therefore, the optimal block site, drug concentration and dose in the IPACK block still needs to be investigated.

Adductor canal block or CACB, combined with IPACK, significantly lowered pain scores within 5 days after surgery compared with ACB or CACB combined with LFA; CACB clearly alleviated pain at 2 weeks and 1 month after surgery [14]. Mariano also showed in a study that continuous regional anaesthetic techniques are preferable to single-injection regional anaesthetic techniques with CACB alone producing continuous analgesia and potentially reducing rebound pain [25]. However, CACB also has related disadvantages, such as follow-up requiring training, increased expenditure of medical resources, and catheter position changes and blockages [7].

In conclusion, it is very important to study the analgesic effect of IPACK after TKA, as it directly affects patient satisfaction and postoperative joint function recovery. The main advantages of IPACK are that it is a promising nerve block method since it provides excellent analgesia and reduces opioid use in combination with other nerve blocks without affecting patient mobility. However, confounding factors in the study must be considered, such as flaws in the study design or analgesic methods that may provide posterior knee coverage. Additionally, other knee surgeries, such as knee cruciate ligament reconstruction and tibial plateau fracture surgery, can be further considered. These studies strongly support the use of IPACK in the multimodal analgesic pathway. Although multimodal analgesia can significantly relieve postoperative pain, preserve motor function, and shorten the LOS, pain reappears with gradual wearing off of the nerve block after discharge, which may increase the probability of readmission analgesia to be required. However, severe rebound pain, hospital readmission due to inadequate pain control, and opioid-related adverse reactions require further development of corresponding protocols, which may lead to the assessment of the feasibility of indwelling catheters in future studies.

This study has its limitations. First, the use of IPACK analgesia combined with other analgesic methods, including ACB, FNB, and CACB; will inevitably have different degrees of heterogeneity which may affect the accuracy of the test results. Second, there is a risk of bias when some articles with low level of evidence are merged. Third, the included studies used general or intraspinal anaesthesia for surgical treatment, which would lead to heterogeneity in the combination. Finally, IPACK, as a newer nerve block method, remains in the exploratory phase in some hospitals, causing further heterogeneity.

## Conclusion

With an increasing need for perioperative services to transition toward an ambulatory model with enhanced recovery, early mobilization, and earlier discharge, IPACK blockade supplementation might be a preferable motor-sparing alternative to TNB with a lower incidence of complications and an increased likelihood of earlier discharge from hospital. Supplementation with IPACK blockade has demonstrated great analgesic effects for early postoperative pain control after TKA, compared to patient-controlled epidural analgesia, and similar to PNB. Moreover, it can significantly reduce postoperative opioid consumption, shorten LOS and improve patient satisfaction. Therefore, IPACK block supplementation as a component of an ERAS protocol for patients undergoing TKA could relieve immediate postoperative pain and encourage a return to daily activities.

## Supplementary Information

Below is the link to the electronic supplementary material.

Supplementary file1 (XLSX 66 kb)

## Data Availability

All data generated or analyzed during this study are included in the Additional file.

## Abbreviations

TKA: Total knee arthroplasty
IPACK: Interspace between the popliteal artery and the capsule of the posterior knee
POD: Postoperative day
POH: Postoperative hour(s)
LOS: Length of stay
ACB: Adductor canal block
PAI: Periarticular injections
CACB: Continuous adductor canal block
FTB: Femoral triangle block
LFCNB: Lateral femoral cutaneous nerve block
SPANK: Sensory posterior articular nerves of the knee
AFCNB: Anterior femoral cutaneous nerve block
RCT: Randomized control trial
VAS: Visual analog scale
NRS: Numerical rating scale
TUG: Timed up and go test
ROM: Range of movement
WMD: Weighted mean difference
SMD: Standardized mean difference
CI: Confidence interval
